# Beyond defence: Immune architects of ovarian health and disease

**DOI:** 10.1007/s00281-024-01021-w

**Published:** 2024-08-12

**Authors:** Maria Victoria Bazzano, Angela Köninger, Maria Emilia Solano

**Affiliations:** 1 Laboratory of Translational Perinatology, University of Regensburg, Biopark 1-3, D-93053 Regensburg, Germany; 2 University Department of Obstetrics and Gynecology, Clinic St. Hedwig of The Order of St. John, University of Regensburg, Steinmetzstr. 1-3, D-93049 Regensburg, Germany

**Keywords:** Prenatal macrophages, Ovary, Follicle reserve, Angiogenesis, Female fertility

## Abstract

Throughout the individual’s reproductive period of life the ovary undergoes continues changes, including cyclic processes of cell death, tissue regeneration, proliferation, and vascularization. Tissue-resident leucocytes particularly macrophages, play a crucial role in shaping ovarian function and maintaining homeostasis. Macrophages crucially promote angiogenesis in the follicles and corpora lutea, thereby supporting steroidogenesis. Recent research on macrophage origins and early tissue seeding has unveiled significant insights into their role in early organogenesis, e.g. in the testis. Here, we review evidence about the prenatal ovarian seeding of leucocytes, primarily macrophages with angiogenic profiles, and its connection to gametogenesis. In the prenatal ovary, germ cells proliferate, form cysts, and undergo changes that, following waves of apoptosis, give rice to the oocytes contained in primordial follicles. These follicles constitute the ovarian reserve that lasts throughout the female’s reproductive life. Simultaneously, yolk-sac-derived primitive macrophages colonizing the early ovary are gradually replaced or outnumbered by monocyte-derived fetal macrophages. However, the cues indicating how macrophage colonization and follicle assembly are related are elusive. Macrophages may contribute to organogenesis by promoting early vasculogenesis. Whether macrophages contribute to ovarian lymphangiogenesis or innervation is still unknown. Ovarian organogenesis and gametogenesis are vulnerable to prenatal insults, potentially programming dysfunction in later life, as observed in polycystic ovary syndrome. Experimental and, more sparsely, epidemiological evidence suggest that adverse stimuli during pregnancy can program defective folliculogenesis or a diminished follicle reserve in the offspring. While the ovary is highly sensitive to inflammation, the involvement of local immune responses in programming ovarian health and disease remains to be thoroughly investigated.

## Introduction

Early prenatal and postnatal life comprises a critical period for organ formation, growth and maturation, which occur in concerted steps [1, 2]. Mammalian sex specification, recruitment of primordial germ cells into the gonads, proliferation and apoptosis, which will give place to the oocytes and spermatozoa, take place largely during gestation [3]. In the case of female individuals, the formation of the so-called ovarian reserve, which refers to overall individual’s oocyte pool, is largely completed prenatally in humans and in the early postnatal days in mice [3, 4]. Moreover, the progenitors of stromal cells that will differentiate into the granulosa and theca compartments in the ovarian follicles are already present in prenatal/perinatal gonads. Follicle granulosa and theca cell layers have crucial steroidogenic functions, as they are a main source of sex hormones during the female reproductive life [3, 4]. Through the secretion of estrogens and progestogens, these cell compartments are not only involved in the female estrous cycle but upon ovulation-induced transformations, they also support early gestation in women and the complete gestation in mice.

New insights highlight the pivotal role of the immune system in regulating reproductive function [5, 6]. Ovarian endocrine roles in young-adult life are possible through the tight interaction with ovarian immune components, critically involved in folliculogenesis, ovulation and corpus luteum formation and regression [7–9]. These processes not only encompass profound tissue remodeling but also the rapid formation of vasculature in discrete ovarian regions. Importantly, macrophages exhibiting hallmarks of M2 activation status are localized in the theca cell layer and in the developing corpus luteum. Here, through the secretion of angiogenic factors, they crucially promote the vascularization required to support the profound steroidogenic activity of these ovarian compartments [10, 11]. Indeed, vascularization not only ensures the influx of oxygens, nutrients, and substrates for sex hormone synthesis, but also their rapid egress into circulation. Interestingly, an altered ovarian immune environment has been observed in conditions with impaired reproductive fitness, such as in polycystic ovary syndrome (PCOS) or obesity [12].

Importantly, over the last decades, accumulating epidemiological data have demonstrated that immune and female reproductive health can be programmed prenatally. Intrinsic and extrinsic insults to pregnancy can challenge maternal physiology, placental function, and fetal organ growth and maturation. In fact, the rapid growth and differentiation of fetal tissues in response to genetic programmes and environmental signals make them particularly sensitive to prenatal insults. According to the Developmental Origins of Health and Disease (DOHaD) theory, such early life insults can permanently influence health and trigger the vulnerability to disease in later life [1, 2, 13].

In this context, it has been recently acknowledged that the prenatal tissue resident immune cell compartment is an important contributor to organogenesis [14]. Hence, we hypothesize that in prenatal life, immune components interact with ovarian processes to program postanatal reproductive health or disease. In the present work we aim to review current literature on the role of immune components in the early development of the ovary. To this end, we delve into aspects of vasculogenesis and innervation of the ovary. These critical developmental milestones can be modulated by immune pathways, and influence early organogenesis and ovarian function in later life. We also revisit the literature on conditions in which prenatal insults may program ovarian dysfunction in adult life and expose the evidence of a possible involvement of the individual’s immunity, as well as current gaps in knowledge.

## Milestones of gametogenesis are achieved prenatally

**Fig. 1:**
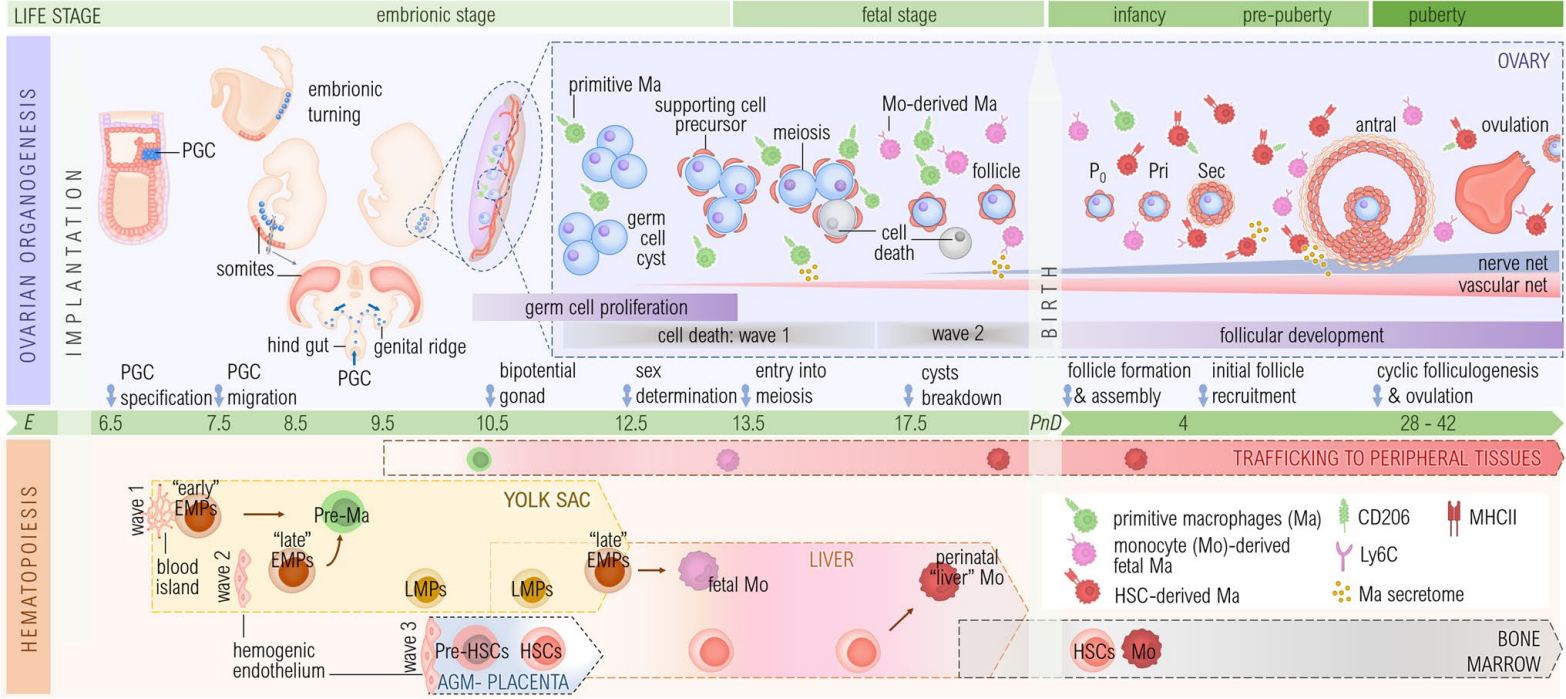
**Ovarian seeding of leukocytes raises questions about their potential contribution to organogenesis in mice.** The upper panel illustrates ovarian organogenesis. Primordial germ cells (PGCs) migrate to the genital ridges at E10.5 and undergo proliferation by synchronous mitotic divisions followed by two later waves of germ cell death. After sex determination (E12.5), PGCs enter meiosis to form oocytes. The vascular (pink) and the neural (blue) innervation begins prenatally. Postnatally, newly formed primordial follicles (P0) start folliculogenesis, developing into primary (Pri), secondary (Se), and antral follicles, leading to ovulation after puberty. Simultaneous to gametogenesis, the ovary is colonized by macrophages (Ma) derived from three hematopoietic waves (lower panel). The first wave, in the yolk sac blood islands, produce “early” erythro-myeloid progenitors (EMPs). “Early” EMPs differentiate into (pre-)primitive macrophages (green) that colonize and expand in the fetal gonad. Also in the yolk sac, the second wave generates “late” EMPs and lymphoid-myeloid progenitors (LMPs). “Late” EMPs seed the fetal liver and differentiate into fetal monocytes. Monocytes colonize the developing ovary to form monocyte-derived macrophages (pink). The third wave gives rise to hematopoietic stem cells (HSCs) in the aorta-gonad-mesonephros (AGM) region, the placenta, and yolk sac, which colonize the liver, and perinatally the bone marrow, to give rise to bone marrow-derived macrophages (red). Macrophage origins and associated markers are color-coded for clarity. Other organs contributing to hematopoiesis are not depicted for simplicity. E: embryonic day; PnD: postnatal day

In human and mice, germ cell specification occurs in postimplantation embryos [15]. In this process, a subset of cells located in the proximal epiblast of the developing embryo [16] are induced to become primordial germ cells by external signals [17]. By embryonic day (E) 7.25 in mice these cells increase in number and are visible as a cluster at the base of the allantois [15, 16] (Fig. 1). Between E9.5 and E10.5, primordial germ cells migrate along the hindgut until they reach and colonize the incipient and yet undifferentiated gonads, called genital ridges [4]. This process occurs in humans until the sixth week of gestation [3, 4]. Interestingly, migratory primordial germ cells differ in terms of their migration traits from many other types of somatic migrating cells, such as fibroblasts, but share many characteristics with both migrating leukocytes and certain types of metastatic cells [18–20]. For example, the stromal cell-derived factor-1 and C-X-C chemokine receptor type 4 (SDF-1–CXCR4) pathway as well as phospholipid signaling through sphingosine-1 phosphate receptor (S1P) and its receptors are important to the migration of these cell types [18, 21]. Upon arrival to the gonad, primordial germ cells enter synchronous mitotic divisions with incomplete cytokinesis. This leads to the formation of so-called germ cells cysts, with sister germ cells derived from a single progenitor connected through stable intercellular bridges [22–24].

By E12.5 in mice and 6–7 weeks of gestation in humans, the yet bipotential gonads commit to a sex-specific fate. In XY embryos the activation of the sex-determining region Y (*Sry*) gene on the Y chromosome which codes for a transcription factor that induces *Sry*-box transcription factor 9 (*Sox9*) expression and a cascade of events that drives the development of gonads into testis. In turn in XX individuals, due to a lack of the *Sry* gene, gonads develop as ovaries. The sex differentiation of gonadal somatic cells dictates the subsequent differentiation of primordial germ cells into oogonia. Of note, germ cells are not required for somatic cell sex differentiation [25]. In contrast to male germ cells, differentiating oogonia present asymmetric accumulation of organelle and cytoplasm over the cyst [22]. The process of sex determination in the gonads will also direct the differentiation of the remaining reproductive organs. In females, in the absence of SOX-9-induced anti-müllerian hormone (AMH), and testosterone produced by the primitive testis, the internal and external urogenital system will continue a female development pathway [4].

By E13.5 in mice, oogonia cease dividing and enter an asynchronous transition from mitosis to meiosis to form oocytes. As oocytes progress through different stages of meiotic prophase I, cyst breakdown begins. During this process, two main waves of cell death of germ cells by apoptosis are described [26, 27]. The first wave of cell death coincides with the entry of oogonia into meiosis (E13.5–E15.5) and the second wave occurs between E17.5 and the day of birth [28, 29]. In humans, only one wave of depletion of the germ cells is reported in the fetal ovary and occurs during mid gestation, with highest apoptosis between weeks 14 and 28 and decreasing towards birth [30, 31]. It has been estimated that the ~ 7 million germ cells in the fetal human ovaries at around week 20 of gestation are decimated to 1–2 million viable oocytes in early neonatal life [31, 32]. Although the most studied type of germ cell death in the ovary is apoptosis, autophagy, necrosis, ferroptosis, or necroptosis may also take place [27, 33].

The oocytes that avoid cell death progress through prophase I of meiosis and initiate primordial follicle assembly. Assembly of the primordial follicle occurs with the encapsulation of individual oocytes by somatic support cells [34]. With assembly of the primordial follicles, the oocytes arrest at diplotene stage of meiosis [3, 4] and initiate the association with proximal somatic cells that later differentiate into two subsets of granulosa cells: mural granulosa and cumulus cells [4, 36]. Perinatally, the ovarian recruitment of theca cells allows the final follicle assembly. Generally, resting oocytes in primordial follicles reside in avascular zones of the ovary. When follicles are recruited to undergo maturation a multilayer blood-follicle barrier forms and protects the oocyte from systemic harmful stimuli and pathogens [35]. The blood-follicle barrier comprises the vascular endothelium and corresponding basement membrane, the thecal cell layer, rich in macrophages, followed by a follicular basement membrane and the granulosa cell layer, in contact with the oocyte [35].

Folliculogenesis starts after birth in mice [28, 29, 31, 37] whereas, in humans the recruitment of resting primordial follicles into the growing follicle population starts already before birth [3]. This gonadotropin-independent follicle recruitment and growth is a continuous but slow process in the prepubertal phase [38]. After puberty, with the initiation of pituitary endocrine stimulation ovulation is achieved. At this time the oocytes complete the first meiotic division with concomitant extrusion of the first polar body [21, 38]. If fertilization with a haploid spermatozoon occurs, the oocyte completes the second meiotic division and extrudes the second polar body [38].

## Multiple systems converge in the female gonad to trace its blueprint

Progression through ovarian organogenesis depends on cell differentiation, proliferation, migration, and tissue remodeling that are guided both by genetic programs and environmental factors. Although the germ cells confer reproductive function to the ovaries, they do not fully determine their morphology [31]. Rather, ovarian somatic cells with simultaneously developing immune, vascular, and neural fetal systems converge in the primitive gonad to trace the blueprint for ovarian structure as follows.

### Subsequent waves of hematopoiesis give rise to immune cells that colonize the tissues during organogenesis

The immune cell fraction is more abundant in male than in female fetal gonads [39–42]. Still, evidence arising for example from the application of high throughput methods to prenatal ovaries such as single cell mRNA sequencing is rapidly accumulating (Table 1). Data from fetal mouse (E14.5), monkey (E84 and E116), and human (20–26 weeks post coitum (WPC)) ovaries [43–45] indicates that the immune compartment shares similarities between species [46] and that macrophages of diverse origins generally account for the main leucocyte subset resident in the prenatal ovary. This relies on the progress of hematopoiesis that, concomitant to early gametogenesis, emerges in three sequential and partially overlapping waves or programs [47] (Fig. 1).

**Table 1:** Published data informing on the immune compartment of the prenatal ovaries

Ovarian immune cells reported	oMa (ovarian macrophages) denomination	tissues	species	life stage	methods	main findings about ovarian leucocytes
blood related cells [43]	-	ovary	mouse	E11.5- 18.5 Pnd 1,5	single cell sequencing	The 8 smallest of 30 clusters gathered endothelial/blood-related cells identified by Cldn5, Car2, Lcn2, and Cx3cr1
F4/80^hi^CD11b^int^ and F4/80^int^CD11b^hi^ Ma [53]	Yolk sac (Moindependent)- Liver (Modependent)derived	ovary	mouse	E14.5-adult	Mouse models: CX3CR1^CreERT2^, Csf1r^Mer−iCre−Mer^ xR26R-EYFP: fate mapping, Plvap^−/−^: ↓liver Mo exit, Ccr2^−/−^: ↓Ly6C^+^Mo, Nur77^−/−^: ↓Ly6C^−^Mo Methods: Ma depletion by antiCSFR1 Ab, single-cell CyTOF	At E14.5 yolk sack-derived Ly6C^−^CD206^+^ oMa are the most abundant leucocyte subset, but are outnumbered at E16.5 by liver-derived Ma
not specified [46]	-	ovary testis	goat, pig macaque, human	various prenatal	single cell sequencing / chromatin accessibility	The proportion of immune cells is relatively stable accross investigated species
TYROB + Ma [44]	-	ovary	macaque	E84 E116	single cell sequencing	E84 and 116 oMa show similar gene signatures and may interact with "Leydiglike" (possible theca progenitor) cells
Stage dependent Ma / other myeloid / lymphoid cells [42]	Tissue repair	ovary testis	mouse macaque human	human: 7–23 WPC	single cell sequencing, spatial mapping, high resolution immaging	Tissue repair oMa coexist in the ovary with T and NK cells. In the testis, Ma are more prominent, some exhibiting exclusive microglia-like traits
Ma and T cells [45]	-	ovary testis	human	4–26 WPC	single cell sequencing	Two clusters of Ma, and two of early T cells differentiate, all with cells from female and male gonads
Stage dependent Ma / other myeloid / lymphoid cells [41]	(Pre-) proangiogenic	ovary testis other	human	4–26 WPC	single cell sequencing, in vitro tube formation assay, imaging	oMa expressing MRC1 (CD206) localized perivascularly and presented an angiogenic profile similar to other tissues, but not the testis

Ma: macrophages, oMa: ovarian Macrophages, Mo: monocytes

The **first hematopoietic wave, or primitive program** starts in mice at E7.0 and at 2.5 WPC in humans [48]. By then, originating from a progenitor in the yolk sac blood islands, the “early” erythro-myeloid progenitors give rise to erythroid, megakaryocyte and macrophage progenitors. Without passing through monocyte intermediates, macrophage progenitors differentiate into pre-macrophages [14]. With the establishment of blood circulation and thus overlapping with the second wave of hematopoiesis, macrophage progenitors, including erythromyeloid progenitors and pre-macrophages, leave the yolk sac and migrate through the bloodstream to colonize all tissues while maintaining their proliferative capacity at least until E12.5 in mice [47–50]. Developing tissues are first colonized by these circulating pre-macrophages, which differentiate into tissue-specific macrophages [14, 50, 51], also known as "primitive macrophages" [47], underscoring the essential role of macrophages in organogenesis.

The **second wave or transient definitive program** initiates within the hemogenic endothelium of the newly developing blood vessels of the yolk sac as from E8.25 in mice and 3.25 WPC in human [49]. The hemogenic endothelium gives then rise to “late” erythro-myeloid progenitors and by E9.5 also to lympho-myeloid progenitors (LMPs), none of them exhibiting the long-term reconstitution potential of HSCs. “Late” erythro-myeloid progenitors generate the first fetal monocytes in the liver, after they colonize it by E9.5. Here, erythro- and lympho-myeloid progenitors continue hematopoiesis so that the liver becomes the main hematopoietic organ until late gestation [14, 52]. The generated monocytes infiltrate the gonads and every fetal tissue, except for the brain, and remain long term to differentiate into tissue resident macrophage populations which will persist at different rates, depending on the tissues [14, 47, 53]. In turn, lympho-myeloid progenitors develop into T and B lymphoid precursors, and later into lymphocytes [47].

The **third wave or definitive program** starts on E10.5 in mice and at 4–5 WPC in human with the emergence of the first hematopoietic stem cells (HSC) from the hemogenic endothelium at the aorta-gonads-mesonephros region [47, 54–56] and beyond E10.5, also at umbilical and vitelline arteries, the placenta and yolk sac. As before the erythro- and lymphoid-myeloid progenitors, HSCs also rapidly seed the fetal liver. At E13.5–14.5 fetal liver is at the peak of hematopoiesis, providing a niche for the expansion and differentiation of hematopoietic stem and progenitor cells [51, 57]. It is unclear to what extent fetal liver HSCs contribute to hematopoiesis before birth [14, 58]. Yet, in the perinatal period, HSC-derived monocytes give rise to a minor population of tissue resident macrophages [47]. Close to birth and in early postnatal life in mice, HSCs egress the liver and seed the maturing bone marrow niche, where they continue hematopoiesis and eventually reach quiescence [14].

#### Following the sequential immune ontogeny, macrophages are the first and main leucocyte subset seeding the ovaries

Macrophages, likely originated from the first and second hematopoietic waves (Fig. 1) [53, 59, 60], are detected around E10.5 in the bipotential XY gonad of mice; however, their presence in the ovaries still needs confirmation. There is general consensus on the importance of macrophages in early life, but not on the nomenclature used across authors (Table 1). Here we refer to mouse macrophages as primitive, when derived from the first wave at the yolk sac, or as monocyte-derived, when originated either in the second wave at the yolk sac or liver, or in the third wave, at the liver. The origin of tissue resident macrophages can be demonstrated e.g. by means of fate mapping models and depletion experiments in mice [60], or cell trajectory analysis in single cell sequencing data [41]. Jokela et al. have used technically sound models to provide a thorough overview of the early life ovarian macrophage compartment. Among prenatal macrophages, generally negative for the major histocompatibility complex class II (MHCII), two populations were described based on the expression of the cluster of differentiation (CD) 11b and the pan macrophage marker F4/80 [53]. In the ovaries at E14.5 CD11b^intermediate (int)^ F4/80^high^ “primitive” macrophages lacking expression of the monocyte marker Ly6C, but positive for the scavenger receptor CD206 are the most abundant population of macrophages. Their origin in the first wave of hematopoiesis is proven by their decline upon antibody mediated-depletion of yolk sac-macrophage progenitors on E6.5 [53]. These observations are in line with those in fetal testis [40, 60, 61], in which tissue CD206 + MHCII- macrophages were abundant and interpreted as exhibiting a M2-alternative activated phenotype.

In humans, early in development (< 9 WPC), at the time that germ cells are migrating to the nascent organ, the ovary immune compartment was to some extent similar to that described in the AGM region [41]. With macrophages representing more than 50% of the total leucocytes, tissue resident cell subsets also included dendritic cells, monocytes and innate lymphoid cells. In these embryonic phases, yolk sac derived-macrophage progenitors and -macrophages are present in the ovary as a small fraction among the major population of pre-macrophages or macrophages with angiogenic features, like in the heart and kidney [41]. This fact, together with the observation that immune cells scattered in the gonad, hilum and extragonadal tissues [42] suggests a rather tissue-unspecific colonization of the ovaries by circulating primitive macrophages and fetal monocytes. Intriguingly, trajectory analysis supported that these early human ovarian macrophages differentiated from yolk sac derived progenitors, without a monocyte intermediary, opening questions about the applicability of the above-described model for immune ontogeny in mice to human early tissue resident macrophages.

Later, on E16.5 in mice, CD11b^high^F4/80^int^ “monocyte-derived” tissue resident macrophages expressing the monocyte marker Ly6C but low CD206 become the most abundant ovarian cell subtype [53]. These CD11b^high^F4/80^low^ macrophages are to a large extent of liver origin, as plasmalemma vesicle-associated protein deficient mice (Plvap^−/−^) mice, with intact yolk sac macrophages (first and to some extent second wave) but defective exit of fetal liver monocytes (second and to some extent third wave) exhibited decreased in CD11b^high^F4/80^low^ macrophages in fetal ovaries [53].

In the ovary, both CD11b^int^F4/80^high^ and CD11b^high^F4/80^int^ macrophages express the gene for colony-stimulating factor 1 receptor (*Csf1r*) [53]. *Csf1r* is widely used in fate mapping studies of macrophages [40, 50, 53]. Pathways downstream CSF1R are crucial for macrophage development and maintenance. Notably, female *Csf1r*^*op/op*^ mice with a naturally occurring Csf1-null mutation [62] show a significant reduction of macrophage in most tissues, including the ovaries [63]. This deficiency leads to disrupted estrous cycles [64], fewer growing follicles [63], low fertility [63, 64] and a low pregnancy rate [64, 65]. A similar reproductive phenotype was observed in *Csf1r*^−/−^ mice [66]. These findings highlight the essential role of CSF1 signalling in ovarian function and fertility.

Between 9 and 23 WPC in humans, a period in which germ cells first proliferate to form cysts and some later undergo cell death, macrophages were variably detected in the ovary. During this time, T cells and NK cells were also abundantly present [41, 42]. This contrasts with the testis, where at all-time points macrophages were the most prominent leucocyte subset. As in mice the phenotype of the macrophages changed with time. After 9 WPC pre-macrophages and macrophages gradually replaced yolk sac counterparts [41]. These subsets were also abundant in the male gonad, where additional macrophages subsets exhibited microglia-like features exclusive of the testis [41, 42]. Similar to mice, ovarian (pre-)macrophages expressed MRC1 gene, that codes for CD206, and presented an angiogenic profile [41]. Some evidence suggests that macrophages could interact for example with theca cell progenitors [39, 53], as they likely interact in postnatal life to promote angiogenesis in this follicle layer. Based on their expression profile, ovarian macrophages are also referred to as tissue repair macrophages [42]. Intriguingly, in *Drosophila* larval ovaries, macrophage-like cells play tissue remodelling roles, by building the basement membrane by deposition of collagen around germ cells, to protect them from excitatory signals that otherwise reduce the reproductive fitness [67]. Notable, their phagocytic/scavenger functions were not thoroughly evaluated for their involvement in e.g. removal of apoptotic germ cells. In other tissues, macrophage mediated phagocytosis of apoptotic cells is key for remodelling processes to reach the final tissue architecture [58].

Clearly, functional studies such as those performed in the testis [40, 60] are still necessary to determine the contribution of macrophages to the stepwise process of ovarian organogenesis. There is also a need for further knowledge of their role in other ovarian developmental processes, including germ cell meiosis and support for stromal cell differentiation and maturation. While a predominant role of macrophages in tissue remodeling and morphogenesis can be proposed, further investigations of their functions in physiological and pathological conditions as well as the exploration of other immune populations are needed.

This prenatal seeding of macrophages significantly contributes to the postnatal immune compartment of the ovary. Also after definitive hematopoiesis is established, macrophages represent an abundant ovarian leucocyte population that accompanies successive tissue changes until organogenesis is completed and gives place to folliculogenesis and later to ovulation. Fetal macrophages traced by labelling of CX3CR1^CreERT2^;R26R-EYFP on E13.5 could be detected in low frequencies in the ovary still at 2 weeks of age. At birth, approximately half of macrophages expressed CD206, although this expression no longer recapitulated a primitive yolk sac origin but rather a tissue resident phenotype [53]. Notably, in the early postnatal ovary, ovarian macrophages remained negative for MHCII, and distributed throughout the stromal compartment and around the follicles in close association with CD144^+^ blood vessels [53]. By 2 weeks of age MHCII^+^ macrophages were first detected and there after markedly increased in the maturing ovaries from mice at least until puberty [53]. Here, similar to bone marrow-derived macrophages, fetal macrophages gained a more activated MHCII^+^ phenotype [40, 53]. In postnatal life macrophages exhibiting an M2-like phenotype are known to be associated with vascular and tissue remodeling during postnatal ovarian development and cancer [39, 40, 45, 48, 61, 68–70]. However, more in depth analysis e.g. of data sets already published (Table 1) could prove useful to understand features specific of ovarian macrophages and their role in ovarian organogenesis.

### Vascularization in the prenatal ovaries coincides with the seeding of macrophages with angiogenic features

In embryonic life the main vascular circuitries are established by the differentiation of stem cells into angioblasts that undergo de novo vasculogenesis. Arising from them, organ blood networks generally form by sprouting and non-sprouting angiogenesis [71]. In E11.5 mice, a time at which germ cells actively proliferate to form cysts, small branches from the mesonephric vessels extent into the 6–8 cell layer stratified epithelium that forms the primordial gonads [72, 73]. This primitive vascular system may constitute a road of access for the early seeding of immune cells into the gonads, as a reciprocal regulation has been described in the undifferentiated testis: failure to form vasculature inhibits macrophage seeding, and macrophage depletion prevents gonad vascularization [60]. In females, this primitive vascular system near the gonadal-mesonephros border will serve as basis for the further angiogenic proliferation and extension of the original branches particularly in the medullar domain of the ovary [72, 74, 75]. This gradual angiogenesis in the ovary contrast to the drastic tissue remodeling in the testis-mesonephros border [72]. Indeed, preexisting vessels in the mesonephros break down, releasing individual endothelial cells that under the influence of cues coded downstream SRY and of tissue resident macrophages [39, 60, 72, 75] are recruited into the testis. As a result, by E12.5 no large vessels or vascular plexuses could be identified near the gonadal-mesonephros border in males [72].

In contrast to angiogenesis, lymphangiogenesis to form the lymphatic vessel network at the ovary starts postnatally in mice [76]. Although proper lymphatic drainage is vital for tissue homeostasis, to date little is known about the contribution of the ovarian immune components to this process as well as about their involvement in ovarian physiopathology.

While not yet fully explored in the prenatal ovary, angiogenic processes typically involve the proliferation and migration of existing endothelial cells, the recruitment of pericytes and smooth muscle cells to stabilize the vessels, and the deposition of extracellular matrix by fibroblasts and mural cells to form the basal membrane. These processes are guided by factors such as vascular endothelial growth factor (VEGF), transforming growth factor β and activation of platelet-derived growth factor receptor β [77]. Alongside cytokines and chemokines, these factors influence endothelial cells to promote angiogenesis [71, 72, 77].

In humans, prenatal tissue pre-macrophages, including most in the ovary, express mRNA for genes that favor angiogenesis, such as VEGFA, IL1B, and CXCL8 [41]. Although not specifically tested in the ovary, fetal macrophages expressing MRC1 (coding for CD206) and CD83 have been shown to secrete soluble factors that promote angiogenesis in vitro [41]. Further, fetal ovarian macrophages expressed adhesion molecules such as ICAM or CD40 that could interact with the endothelium [41] or with theca cell progenitors [44] to promote angiogenesis. Further evidence of such an interaction is the observation of human ovarian macrophages, particularly those expressing MRC1 preferentially localized at the perivascular space, as opposed to other leucocytes scattering through the tissue [41]. This regulation between fetal macrophages and endothelium might be reciprocal, as supernatants from cultured endothelium also induced angiogenic genes in macrophages [41].

Based on their mRNA expression profile, ovarian and subtypes of testicular macrophages have also been referred to as tissue-repair macrophages [42]. In general, macrophages can modify the extracellular matrix e.g. by secreting matrix metalloproteinases (MMPs) that degrade the extracellular matrix and create a permissive environment for endothelial cell migration during angiogenesis [74]. Of note, although MMP9 expression was a hallmark of macrophages in the testis, it was not expressed by the ones in the ovary [42].

Taken together, accumulating information from mRNA expression analysis in human tissues support a role of macrophages in angiogenesis in the ovary [41, 42]. Such angiogenesis involves less dramatic tissue remodelling than in testis [72], where the upstream regulation of macrophages has been already experimentally confirmed [40]. In contrast, direct evidence, and mechanisms of macrophage-induced angiogenesis in the ovaries are missing and require experimental validation, e.g. in mouse models. Importantly, with the initiation of folliculogenesis, the vascular net accompanies the changes in the ovary, by irrigating follicles at the level of the outer layer, composed by theca cells [76]. This involves cyclic processes of angiogenesis during follicular development, and, when ovulation is established, in corpus luteum development, remodeling, and resorption [10]. In the cycling female, these processes are under the close regulation of ovarian macrophages [10].

### Innervation of the female gonads

Postanatally a complex interplay between the autonomic (sympathic and parasympathic) nervous system, hormonal signals, and sensory nerves [76] contributes to the control and coordination of ovarian function to an extent that their defect can disrupt ovarian health. However, specific mechanisms of how ovarian and neuronal cells synergize to regulate the ovary remain poorly studied [78, 79].

Similar to most of the peripheral nervous system, the ovarian nervous system is derived from the neural crest progenitor cell population, which colonizes target organs during prenatal development [79, 80]. As the vasculature, peripheral innervation is a sexually dimorphic component of the gonad involved in its organogenesis. The presence of neural projections within the ovarian medulla has been identified in fetal and adult life in the mouse and human ovary [80]. Already by E15.5 neural crest cell-derived projections are observed entering the dorsal mesonephros of both male and female fetal mice [72, 79]. At E16.5 neural crest cell-derived progenitors colonize the dorsal face of the ovary and differentiate into neurons and glia. In contrast, at E16.5 innervation in the male reproductive complex is restricted to the epididymis and vas deferens, never reaching the interior of the testes due to the expression of avoidance signals [79]. From E18.5 onward, ovarian innervation gains complexity to give rise to a dense neural network within the developing ovarian medulla [72, 79]. Recruitment of neural crest-derived neurons and glia into the ovary coincides with critical patterning events during ovary development, including rotation of the cortex to the ventral surface of the ovary, establishment of the hilus, germ cell cyst breakdown [22], primordial follicle formation [80], and activation of the first wave of growing follicles soon after birth [78, 81]. In mice, ovarian theca cells are originated from Wt1^+^ ovarian precursor cells and Gli1^+^ mesenchymal cells migrating from the mesonephros between E17.5 and postnatal day (PnD) 5 [79, 82]. The migration pattern resembles ovarian innervation during development, but whether they use the same guidance cues is unknown. It is also unknown whether the remodeling of the nerves is guided by macrophages, as it occurs in the brain with the microglia [14, 58]. As shown in adulthood, peripheral nerves house specific resident macrophages which to some extent originate prenatally and hold self-renewal capacities [14, 83]. Whilst recent reports indicate that peripheral nervous system macrophages, like brain microglia, are critical for remodeling in homeostasis and inflammation [83] the literature has not yet described such a population and their role in fetal ovarian innervation.

Postnatally, in the prepubertal ovary, each growing follicle is innervated by a single neuronal fiber [84]. These projections of nerve fibers reach the theca cell layer of the follicles [84], rich in macrophages and blood capillaries, where a coupling between nerve activity and modulation of the blood flow has been proposed. Here, ovarian innervation may be involved in stimulating theca or smooth muscle cells during follicle growth and ovulation [79, 85]. To date, a role for macrophages in the seeding, differentiation, and elongation of the neural crest progenitor cell in the fetal ovary, or later, in the innervation of the developing follicles has not been yet investigated. In other tissues, e.g. in dermis, the populations of macrophages associated with blood vessels and nerves differentiate with regards to their origin and phenotype [14] with nerve-associated macrophages facilitating processes of regeneration or remodeling [14]. The mechanisms linking vascular development to ovarian innervation, as well as the interplay between immune populations such as macrophages and ovarian innervation, remain a challenge to be solved.

## Early programming of ovarian dysfunction in human and rodents

Despite the progress in understanding the fine networks regulating ovarian function, subfertility of unknown origin affects a significant fraction of the female population [86]. Upon the formulation of the DoHaD hypothesis [13], research has questioned the transgenerational origins of ovarian dysfunction. Given the delayed emergence of reproductive symptoms and the influence of adverse postnatal environmental factors on the manifestation of fetal programming effects [87], comprehensive investigations are constrained by the need of clinical data from population-based cohorts or studies entailing long-term participant follow-ups. In this section, we examine the evidence arising from the investigation of maternal conditions affecting pregnancy, namely intrauterine growth restriction, the use of assisted reproductive technology, acetaminophen intake, maternal stress perception, obesity, and polycystic ovary syndrome, which may hold consequences in the programming of reproductive dysfunction (Table 2). In this context, clinically relevant animal models have proved crucial for confirming the impact of prenatal insults on postnatal reproductive function and hold a potential application for exploring potential mechanisms or therapies (Table 3).

**Table 2:** Maternal conditions during gestation and consequences for the offspring

Maternal condition	Polycystic ovary syndrome (PCOS)	APAP and NSAIDs intake	Obesity	Infection Immune activation	Stress perception	Intrauterine growth restriction Small for gestational age	Assisted reproductive techniques
Life stage assessed	puberty adulthood	perinatal	perinatal	perinatal	puberty adolescence adulthood	prenatal puberty	puberty adolescence
AGD	↑	NR	NR	NR	NR	NR	NR
Puberty	early onset	early onset	early onset	NR	early onset	= or early onset	early onset
Neurosex hormones	↑ LH ↓E2 ↑T ↑DHEAS	NR	↓DHEAS ↓ E2	NR	↓T ↓DHEAS	= LH = FSH = E2 ↑↑ = T	↑LH ↑DHEAS
Follicular reserve Ovary features	↑ AMH ↑ ovary size: polyfollicular	NR	= AMH = follicle count	NR	= AMH ↑follicle count ↓PCOM risk	= AMH ↓germ cells ↓ovary size ↑PCOM risk oligo/ anovulation	NR
Risk of infertility	↑: related to PCOS symptoms	NR	NR	NR	= or ↑ risk	no known association	NR
Immune features*	Blood: chronic low grade inflammation (e.g ↑leukocytes ↑CRP ↑IL-6) Follicular Fluid: ↑leukocytes ↑M1like macrophages ↑chemokines ↑NFκB ↑cytokines ↑angiogenic factors	*cord blood:* ↓HSCs	*cord blood*: ↓eosinophils, ↓CD4 T cells, ↓ monocyte, ↑DCs responses to tolllike receptor ligands, ↑IFNα2, ↑IL-6; ↑TNF-α, ↑IL- 1; ↑macrophages	Amniotic fluid and cord blood: changes in metabolites and methylation consistent with inflammation	Cord blood: cytokines upon stimulation: ↑ IL-1β, IL-6, ↑ IL-8, IL-4, IL-5 changes in DNA methylation	*Cord blood:* ↑GCSF, ↑IL-12, ↑IL8, = IL-1a, = IL-6, = IL-10, = IP-10, = MCP-1, = MCP-3, = MIP-1a, = TNF-α	*blood*: ↑IFN-γ; ↑IL-4; ↓IFN-γ/IL-4, main immune cells subset unchanged
Risk for disease*	↑ CVD, ↑ asthma, ↑ allergies	↑ CVD, ↑ asthma	↑ CVD, ↑ asthma	↑ CVD, ↑ asthma, ↑ eczema	↑ asthma ↑ allergies	↑ CVD, ↑ asthma, = allergies	↑ CVD, ↑ asthma, = allergies
References	[151, 152, 153, 154, 155, 170, 171, 172, 173, 174, 175, 176, 177, 178]	[123, 128]	[135, 136, 179, 180, 181, 182, 183, 184, 185, 186]	[187]	[104, 107]	[89–95, 98]	[113, 114, 117, 188]

*Information may include findings also in male offspring, as most publications did not detail the gender of the subjects. Abbreviations: NR: not reported, “↑”: increase, “↓”: decrease, “ = ”: unaffected, anti-müllerian hormone: AMH, testosterone: T, dehydroepiandrosterone-sulfate: DHEA-S, estradiol: E2, luteinizing hormone: LH, Follicle-stimulating hormone: FSH, anogenital distance: AGD, Interleukin: IL, C-reactive protein: CRP, nuclear factor kappa B: NF-κB, monocyte-chemotactic protein: MCP, Chemokine: C-X-C motif ligand: CXCL, C-X-C motif chemokine receptor: CXCR, Macrophage inflammatory protein: MIP-1α, dendritic Cells: DCs, Interferon: IFN, polycystic ovary morphology: PCOM, Granulocyte-Colony Stimulating Factor: G-CSF, cardiovascular disease: CV

**Table 3:** Rodent in vivo models of challenges to human pregnancy and consequences for fertility

Rodent in vivo models of challenges to human pregnancy		Maternal polycystic ovary syndrome (PCOS)	Use of over the counter analgesics: acetaminophen (APAP)	Diet induced obesity	Maternal immune activation	Maternal stress exposure	Intrauterine growth restriction
Species		rat / mouse	rat / mouse	rat / mouse	rat	rat / mouse	rat
Intervention		prenatal androgenization: Testosterone (T), dihydrotestosterone (DHT), AMH	APAP (50—350 mg/kg/d, gavage/ i.p.)	26–60% kcal high fat, high sugar diet (HFD/HSD)	Lipopolysaccharide (LPS) 50 μg/kg [161, 162, 164, 165, 197] or 18 μg/kg, i.p. [161]	various paradigmes of social, extreme temperature, light or restrain exposure [108, 109, 198–200]	Hypoxia due to uterine artery ligation, or exposure to hypoxia chamber
Duration and time of the intervention		3–5 d last wk of pregnancy	1, 7–25 d (starting 2 or 3 wk of pregnancy)	various convinations of HFD/HSD before, in pregnancy, lactation + offspring HFD/HSD	1. day challenge in early, mid, or late pregnancy [161-163, 198] 2. days on PND3-4 (neonatal) [164, 165]	full or late pregnancy (~ 7d)	full [201] or 3–5 d in last wk of pregnancy [96, 202]
Endocrine outcomes in the F1 offspring	Anogenital distance	↑ [189, 190]	-	-	-	=	-
	Puberty onset	*delayed *[192]	-	-	delayed [161, 162]	*delayed *[109]	*delayed *[202]
	Estrous cycle	*altered *[191]	-	-	-	*longer *[108]	*-*
	Sex hormones (serum)	↑ T ↑ LH:FSH; ↑ GnRH/LH pulse frequency [188, 193, 194]	-	*contradictory LH,* *FSH, E2 *[140, 141]	*Pnd 5–14: ↑ E2 ↑ T from Pnd14 to* *Pnd30; ↓LH at Pnd14; ↓E2 and T at Pnd80; Pnd 45-* *50: ↓FSH* [161, 162, 164]	↓E2 [108, 109, 200]	-
Ovarian outcomes in the F1	ovarian reserve	-	prenatal:↓ PGC; Pn: ↓ primordial follicles [131, 132]	↓ o = primordial follicles [142–144]	↓primordial follicles [164]	= or↓ primordial follicles [108, 109, 198]	↓primordial follicles [201, 202]
	AMH	↑ [191, 192]	↓ in ovarian follicles [103, 196]	-	↓	-	*↓ *[96, 201]
	follicular growth	*↑* = *↓ (pre)antral follicles* [152, 189, 192, 193, 195]	*↓ follicles *[131]	↓preantral / antral follicules [141, 143, 145]	↓ preantral follicles [162, 163]	= or ↓ preantral/ antral follicles, = or ↓ corpora lutea [108, 109, 198]	↓ total, (pre)antral follicles, = corpora lutea [96, 202]
	atresia	↑	-	↑ [140, 145]	↑ [162, 163]	-	-
	vascular net/ innervation	-	-	*↑ vascular congestion, perivascular oedema *[143]	-	*Neonatal ovary: ↓* *NGF* *Pubertal Ovary: ↓* *NE *[109]	*contradictory* *Tgfb2 *[96, 201]
	immune traits	-√	-	↑ NF-κB [143, 144]	*↑ TNF-α, TGF-β1* [165, 197]	-	
Other immune manifestations in the F1*		√	√	√	√	√	√
Signs of maternal immune activation*		√	√	√	√	√	√

Text in *italics* indicates that the evidence requires confirmation, either because the reported results are contradictory, or because they have not yet been replicated. In this table, assisted reproductive techniques were not included as a challenge as its consequences on reproductive fitness of the offspring are unknown. Abbreviations: “- “: not reported, “↑”: increase, “↓”: decrease, “ = ”: unaffected, anti-müllerian hormone: AMH, testosterone: T, estradiol: E2, luteinizing hormone: LH, follicle-stimulating hormone: FSH, nuclear factor kappa B: NF-κB, Interferon: IFN, Transforming growth factor beta 1: TGF-β1, norepinephrine: NE, neural growth factor: NGF, “yes”: √

**Intrauterine growth restriction (IUGR)**, constitutes a serious and prevalent condition in which the growth trajectory of the fetus is below its potential [88]. IUGR is associated with increased inflammation in the mother and offspring, as evidenced e.g. by enhanced levels of cord blood G-CSF, IL-12, and IL-8 in this population [89]. Ovarian development could be targeted by this perinatal immune activation, as a higher incidence of PCOS, and potentially reduced fertility [90–95] have been proposed in girls who suffered from IUGR. There are indications that inadequate intrauterine growth is linked to reduced ovarian volume and primordial follicles [96] early in life. In line with these observations, various animal models applying intrauterine hypoxia to simulate placental insufficiency and IUGR (Table 3) resulted in reduced primordial follicles and AMH [97]. Later in life, compensatory mechanisms may be at play, so that ovulation and fertility of the females are preserved in the long term [97, 98]. Further research is needed to clarify these complex dynamics and to explore the contribution of the immune alterations in the context of IUGR to the programming of ovarian function. Clearly, a difficulty in the context of the human clinical condition, is the diversity of factors that can be associated with IUGR symptomatology. This includes pregnancy complications such as preeclampsia or challenges such as prenatal stress, assisted reproductive techniques (ART), or exposure to xenobiotics that have been associated with an enhanced risk for IUGR in the offspring [99]. Due to the diversity of intrauterine processes in these conditions, it is also expected that they may affect differently the fetal immune and reproductive development, as described below.

**Maternal stress perception during pregnancy** can significantly target the offspring’s neuroendocrine development and trigger their risk for allergies in later life [100, 101]. Exposure to prenatal stress was associated with increased serum levels of IL-1β, IL-6, IL-8, IL-4, and IL-5 at birth, as well as to deregulated cytokine secretion by cord blood cells in vitro stimulated with triggers of innate and adaptive immunity [102, 103]. Importantly, large population-based studies have provided evidence that maternal stress exposure was associated with a slightly earlier puberty onset [104], higher antral follicle counts, unaffected circulating AMH [105], but lower testosterone and androstenedione [106] in adolescence than in the matched reference population. Intriguingly, maternal exposure to stressful life events resulted in lower prevalence of polycystic ovary morphology, but not PCOS in girls [106]. Despite these mild changes, the long-term follow up of a population of more than 660 thousand women indicated a higher risk for infertility in women exposed prenatally to stress [107], which might reflect trends towards lower rates of follicular maturation and ovulation observed in animal studies [108, 109]. To date, short and long-term changes in the ovarian innervation have been described in rodents prenatally exposed to stress [108]. However, further cues to explain these reproductive alterations are still missing and their possible association to immune changes in prenatal and postnatal life offer mechanistical pathways to consider.

**The use of ART** has recently raised concerns about potential risks for the health of the offspring in later life. Common methods of ART include intrauterine insemination, in vitro fertilization, and intracytoplasmic sperm injection [110, 111]. Not only are ART conceived individuals at increased risk for obstetric complications such as IUGR [112]; but also, the environmental conditions during ART procedures, e.g. hormone administration, gamete, and embryo manipulation, may pose a risk for long-term alterations in the health of the exposed individuals [111]. In fact, children born after ART exhibited altered immunity, with increased rates of immune-related diseases and elevated circulating interferon-γ and IL4 [113] than children born after natural conception [101, 114–117]. In line with this, immune alterations in mouse models of ART-conception were also reported [111, 118, 119]. Nonetheless, it is yet unclear whether such immune activation may affect female ovarian function. In humans this interconnection is particularly elusive, as parental causes of infertility and maternal health may significantly influence outcomes [120]. Of note, insights from a register-based study that evaluated 122. 321 ART-conceived and 6 0.576. 410 non-ART singletons [114] concludes that girls born after ART had more diagnoses related to early puberty that the ones born after natural conception. In conclusion ART seems to trigger detrimental immune responses in the offspring, albeit the effect on the ovarian development needs urgent investigation.

### Acetaminophen intake

The recognition that exposure to chemicals during pregnancy can affect offspring’s development with long-lasting effects on the reproductive health has raised concerns on the use of medication in pregnancy. Currently, N-acetyl-para-aminophenol commonly called acetaminophen or paracetamol, is the most frequently used over-the-counter analgesic to treat fever and pain during pregnancy [121–124] often also as a self-administered treatment without clinical supervision [124]. Although safer than other pain medications, acetaminophen can cross the placenta [121, 125, 126], and its toxicity is enhanced in pregnancy due to decreased liver drug-metabolism [121, 125]. For these reasons, addressing possible effects of acetaminophen has become a matter of outmost urgency. Studies in humans and rodents demonstrate that maternal acetaminophen intake can lead to signs of immune activation in the mother, placenta and offspring [125, 127–129]. Fetal immune ontogeny appears particularly vulnerable as HSCs were reduced in infants’ cord blood [123] and in mouse fetal liver [129] after maternal acetaminophen intake during pregnancy. Maternal acetaminophen exposure in rodents also influenced the prenatal gonadal development in male and female offspring [127]. Whilst comparatively fewer studies evaluated the effects on female offspring (Table 2, 3) acetaminophen exposure in utero decreased the number of ovarian germ cells [130–132] and AMH expression [130]. These observations hold truth regardless of the various time of exposure during all, mid or late pregnancy, pinpointing mid-late gestation as especially vulnerable periods to target the follicular reserve. In human ex vivo fetal ovary cultures, acetaminophen directly reduced steroidogenic function [133] and the density of small, immature germ cells [133] also altering their expression of differentiation markers [134]. So far, cohort studies only described earlier puberty onset in females exposed prenatally to acetaminophen [128]. Taken together, while the evidence on the deleterious effects of acetaminophen on germ cells and immunity is robust, potential associations between the local effect of acetaminophen on ovarian tissue resident immune cells and formation of the ovarian reserve require further investigation, particularly in humans.

**Maternal obesity during pregnancy** is also becoming increasingly prevalent [135], as are the complications associated with this condition. Maternal obesity-associated placental dysfunction can result in small-, or, more frequently, on large-for-gestational-age neonates. Both conditions left children at an increased risk of metabolic, inflammatory, and chronic diseases later in life [136, 137]. Enhanced oxidative stress and inflammation [103] may explain the exacerbated immune activation (Table 3) with e.g. increased IFNα2, IL-6, TNF-α, and IL-1 in the cord blood of neonates born to obese mothers [137]. The impact of maternal obesity on female offspring’s fertility is not clear in humans, with no effects on the follicular reserve or fertility reported (Table 2). In stark contrast, rodent models of dams fed high fat/sugar diet (HFD/HSD) before and during pregnancy indicated that female offspring enter puberty early, tend towards a reduced ovarian reserve and present a disrupted ovarian and estrous cycle [138–147] with upregulated ovarian NF-kB expression [143, 144]. In the ovary, NF-kB is known to promote immune and inflammatory responses and to regulate granulosa cells in the formation of ovarian follicles [143, 144], which may offer insights into the mechanisms leading to decreased follicular reserve in these females. Despite these insights, in depth investigation of early immune regulation in the ovary is essential to shed light on potential downstream processes such as the development of vasculature and innervation, and their contribution to an impaired follicular ovarian reserve upon prenatal maternal obesity. Of note, HFD during pregnancy suffices to affect offspring fertility but not to induce maternal obesity. Instead, it seems likely that nutrients and metabolites originated by the diet and/or changes in the maternal microbiome are important mediators and effectors of these reproductive changes other than increased BMI. Shall this be the case, it may provide an explanation for the variability observed in outcomes from women with high BMI compared to HFD rodent models.

**Polycystic ovary syndrome (PCOS),** often associated with obesity and metabolic derangements, is the most frequent neuro-endocrine disorder among women of reproductive age [148]. PCOS women present high levels of ovarian androgen production, ovulatory disorders, and/or ovarian follicular cysts. Intriguingly, PCOS is associated with low-grade inflammatory symptoms which also manifest in the ovary by means of exacerbated activation of the NF-κB pathway, cytokines, adhesion molecules, and chemoattractant factors [149]. There is a high heritability of PCOS features from mothers to daughters, that exceeds genetic inheritance and is consistent with fetal programming effects [150]. Such features include elongated anogenital distance (AGD) [151, 152], elevated circulating androgens and AMH [151, 153–155], as well as polycystic ovary morphology [150, 152, 155]. PCOS-like characteristics can also be reproduced in rodent and primate models by prenatal androgenisation [152] (Table 2), pinpointing the hyperandrogenism in pregnancies as the main driver of the long-term changes in the offspring, for example at the hypothalamus / pituitary level [152]. However, PCOS and prenatal androgenisation models also induce signs of maternal immune activation [153]. The exacerbated immune status of PCOS women persists during pregnancy [156]. E.g. circulating IL-1β, IL-2, IL-6, IL-12, CRP, IL-8, and TNF [156] are particularly increased in the first trimester of pregnancy, a crucial time for offspring's ovary and immune development. Hence, important questions about how this cytokine milieu may contribute to the seeding of macrophages in the ovary, its development and dysfunction later in life remain open. In fact, ovarian inflammatory macrophages appear consistently increased in women and mice with polycystic ovaries, implying a likely contribution to the ovarian pathology [149, 153]. In line with this, excessive vasculature and VEGF levels are described in polycystic ovaries [157]. Ovarian innervation may also be involved in ovarian derangements as follicular cysts have been observed in transgenic mice overexpressing nerve growth factor in the theca cell layer [158]. As ovarian vascularization and innervation initiate in prenatal life, and might be influenced by macrophages, it is tempting to hypothesize that they might be additional targets of the dysregulated prenatal development.

**Maternal immune activation**, referring to the activation of inflammatory pathways e.g. due to viral and bacterial infection, results in the release of cytokines and chemokines that can cross the placenta. Maternal immune activation is a condition well known to alter offspring’s neuroendocrine and immune responses, often in a permanent manner [159, 160]. Bacterial lipopolysaccharide (LPS)-triggered maternal immune activation during mid or late pregnancy in rats further resulted in smaller female offspring with delayed puberty onset, lower sex hormone secretion [161, 162], and fewer ovarian follicles [163], often forming follicular cysts [162] (Table 3). LPS exposure coincided either with the time of sex specification and germ cell proliferation in mid pregnancy, or later, with the waves of apoptosis prior to germ cell cyst break down. However, it is unknown how systemic or ovarian inflammation affected the process of gametogenesis, and whilst an involvement of the ovarian immune compartment is very likely, it has not yet been investigated.

### Many conditions, one mechanism?

Considering the outcomes presented in Table 2, the reproductive features in human cohorts exposed to the reviewed prenatal conditions were variable. In girls, the most frequent observation was an earlier puberty onset than in the reference population. In contrast, in rodents the puberty onset was mostly unaffected or delayed (often linked to impaired follicle maturation) upon prenatal challenges. As puberty onset occurs by similar mechanisms in mice and human, primarily responding to hypothalamus and pituitary activation, and heavily influenced by postnatal metabolic and environmental factors, the origin of the mismatch between species is unknown and requires further investigations. As referred to in Table 3, in rodent models of challenges to pregnancy alterations in ovarian cyclicity and a reduced ovarian reserve were often observed. Still in human cohorts menstrual cycle was not reported and no significant changes in the ovarian follicle reserve were observed. This might reflect the difficulties to assess the ovarian reserve in humans, which generally relies on circulating AMH or number of developing follicles. By not directly assessing resting follicles, these measurements could be subject to error. Intriguingly, a reduced follicular ovarian reserve does not result in necessarily subfertility in young individuals [164]. For example, female rats with a low ovarian reserve were fertile although their pups experienced impaired development, growth, and fitness upon pregnancy [165]. This is not surprising, as many of the discussed challenges to pregnancy transmitted the reproductive phenotype over generations. In the affected individuals, a reduced ovarian reserve can lead instead to a precocious depletion of oocytes and a shortened reproductive life span for females, with later consequences for bone and vascular health [166]. To date, investigations addressing the incidence of premature ovarian insufficiency, ovarian aging, or menopause in females affected by prenatal challenges, which in humans would require a particular long follow up time of study participants, are missing.

Intriguingly signs of maternal immune activation were observed in most challenges to human pregnancy summarized in Table 2 and reproduced in rodent models (Table 3). Also, alterations in cytokines or chemokines were often detected in cord blood of infants born upon prenatal challenges. Hence, the reduced ovarian reserve upon prenatal insults in rodents not only pinpoints the vulnerability of germ cell formation to environmental cues. Experimental and clinical observations on factors that can influence the ovarian reserve also puts a spotlight on immune reactions as a common denominator of prenatal challenges (Table 2, 3). Likely maternal and infant immune activation in human and rodents experiencing metabolic or endocrine conditions or challenges to pregnancy were of a smaller magnitude than those elicited by infections or LPS intervention. Regardless, these observations open unanswered questions about the role of maternal immune activation as a common pathway to mediate fetal programming of female reproductive health.

The postnatal ovary is in fact highly vulnerable to inflammation. Although a rarely described event, Mumps or cytomegalovirus tropism and infection to the ovary can lead to acute inflammation (oophoritis), tissue necrosis including follicle depletion, and premature ovarian failure [167, 168]. Evidence on whether equivalent congenital infection of the offspring during pregnancy can also affect the follicular reserve is still elusive. However, life stages in which the assembly of primordial follicles is still ongoing, such as the early postnatal period in rodents (~ second trimester of pregnancy in women) [3, 4], and hence prior to the formation of the blood-follicular barrier [35], appear particularly vulnerable to inflammation [165]. In neonate rats, the administration of LPS upregulated systemic inflammatory cytokines, and was sufficient to induce ovarian NFκB pathways, local inflammation resulting in oocyte depletion, and impaired ovarian reserve [165]. In this context as well as in offspring affected by prenatal insults, it remains to be investigated whether ovarian macrophages, as the main tissue resident cell subset at the time, are responsible for sensing and reacting to such signals to amplify inflammation [169], and what are the consequences for apoptosis of germ cells, vascularization, and innervation of the gonad prenatally and during the female fertile life.

## Final remarks

Despite significant progress in understanding the contribution of fetal macrophages to early organogenesis, research on their role in ovarian morphogenesis lags behind. Due to macrophage sensitivity and plasticity to the environment, their dysregulation in the context of prenatal insults affecting the ovary is also expected. Whether tissue resident yolk sac and/or fetal monocyte derived macrophages enhance or ameliorate the impact of insults on the ovary, including the programming of impaired function and shortened reproductive life remains to be investigated. The ongoing advancements in the development of mouse models and technical methods that enable the analysis of small sample sizes and cell numbers, as is the case with fetal ovaries, promise exciting opportunities to bridge these gaps in knowledge in the near future.

## Data Availability

There is no additional data associated with this manuscript.
